# Soil Bacterial Diversity and Community Composition Across Contrasting High-Andean Sites in Candarave, Southern Peru

**DOI:** 10.3390/microorganisms14102356

**Published:** 2026-10-15

**Authors:** Pablo Franco-León, Liduvina Sulca-Quispe, Alex Yaja, Danuzia Batista Da Silva e Souza, Alonso R. Poma Ticona, Fabyano A. C. Lopes

**Affiliations:** 1 Research Group on Arid Zones, Deserts, and Climate Change (ADERIZA-UNJBG), Universidad Nacional Jorge Basadre Grohmann, Tacna 23003, Peru; pfrancol@unjbg.edu.pe (P.F.-L.); ayajac@unjbg.edu.pe (A.Y.); 2 Mycology Laboratory, Science Faculty, Universidad Nacional Jorge Basadre Grohmann, Tacna 23003, Peru; lsulcaq@unjbg.edu.pe; 3 Microbiology Laboratory, Universidade Federal do Tocantins, Porto Nacional 77500-000, TO, Brazil; danuzia.batista@professor.to.gov.br; 4 Enzyme Biotechnology Research Laboratory, Science Faculty, Universidad Nacional Jorge Basadre Grohmann, Tacna 23003, Peru

**Keywords:** soil microbiome, bacterial diversity, high-Andean drylands, aridity trend, land use, 16S rRNA gene amplicon sequencing, microbial ecology, southern Peru

## Abstract

High-Andean drylands are environmentally heterogeneous ecosystems, yet soil bacterial communities in southern Peru remain poorly characterized. This exploratory study examined bacterial communities in 23 soil samples collected from five sites in Candarave, Tacna, representing herbaceous–shrubland, succulent shrubland, and agricultural environments that also differed in elevation, soil pH, moisture, and historical aridity descriptors. Bacterial communities were characterized by 16S rRNA gene amplicon sequencing. Actinobacteriota, Chloroflexota, and Proteobacteria predominated across the sampled sites. Alpha diversity differed among sites (Kruskal–Wallis, *p* ≤ 0.009) and was highest at the two agricultural sites (P4 and P5; Shannon index, 11.33 ± 0.34 and 11.35 ± 0.17) and lowest at the herbaceous–shrubland site P1 (9.52 ± 0.75). ANCOM-BC indicated higher abundances of Azospirillales at both agricultural sites and of *Blastococcus* and *Solirubrobacter* mainly at P4, whereas *Candidatus Udaeobacter* and *Conexibacter* were more strongly represented at P3, the succulent shrubland site. Unweighted UniFrac analysis showed marked differences in bacterial community composition among the five sampling sites (PERMANOVA, pseudo-F = 4.67, *p* = 0.001), while multivariate dispersion did not differ significantly (PERMDISP, F = 0.686, *p* = 0.153). These results provide a descriptive baseline for soil bacterial diversity in an understudied high-Andean region and document substantial community heterogeneity among sites differing simultaneously in environmental conditions, vegetation, and land use.

## 1. Introduction

Soil bacterial communities contribute to organic matter transformation and nutrient cycling, while their composition is closely associated with the physicochemical conditions of the soil environment. Rather than responding to a single environmental variable, bacterial assemblages are influenced by differences in water availability, pH, nutrient status, organic matter, and vegetation, which together shape the conditions under which microbial communities develop [1]. These relationships become particularly relevant in drylands, where water limitation and associated changes in soil properties can be accompanied by marked shifts in microbial diversity and community composition [2,3]. Such environmental heterogeneity is commonly reflected in spatial differences in bacterial diversity and community composition across dryland soils.

High-elevation drylands are characterized by limited water availability, large temperature fluctuations, intense solar radiation, and low nutrient availability, conditions that can strongly constrain soil microbial communities. Across dryland ecosystems, increasing aridity has been associated with changes in microbial diversity and community composition, although these responses vary among regions and local environmental settings [2,4]. In the Central Andes, high-altitude soils harbor distinctive bacterial assemblages under oligotrophic, desiccating, and high-radiation conditions [5,6]. Along mountain gradients, changes in climate also occur alongside variation in vegetation and soil properties. In an arid mountain ecosystem, for example, bacterial diversity and community composition varied with precipitation, vegetation characteristics, and soil properties, including plant cover, plant diversity, and available nitrogen [7].

Vegetation and land use can further contribute to spatial variation in soil bacterial communities by modifying resource availability and local soil conditions. Differences in plant cover influence organic matter inputs, root-derived compounds, soil moisture, and nutrient availability and have been associated with changes in bacterial community composition across contrasting vegetation types [7,8]. Agricultural management introduces additional changes through practices such as tillage, irrigation, fertilization, and crop establishment, which can alter soil properties and bacterial community structure [9,10]. Similar patterns have been reported in the Peruvian Andes, where traditional potato tillage systems differed in bacterial diversity and evenness, although richness did not differ, and soils under full tillage showed greater heterogeneity in bacterial community composition [11].

Despite these advances, information on soil bacterial communities in the southern Peruvian Andes remains limited, particularly across landscapes where natural vegetation and agricultural areas coexist under contrasting environmental conditions. Most available studies from high-Andean systems have focused on particular habitats or management settings, including native vegetation in the Chilean Andes and traditional agricultural soils in the central Peruvian Andes [6,11,12]. Candarave, in southern Peru, contains high-Andean natural and agricultural landscapes characterized by marked differences in elevation, vegetation cover, soil conditions, and historical climatic variability. The province is also characterized by recurrent drought and ongoing desertification processes, which add further environmental heterogeneity to this landscape [13,14]. However, how soil bacterial communities vary among these contrasting high-Andean sites remains poorly documented.

In this study, we characterized soil bacterial communities across five high-Andean sites in Candarave, southern Peru, representing herbaceous–shrubland, succulent shrubland, and agricultural environments that also differed in elevation, soil pH, moisture, and historical aridity-trend estimates. Using 16S rRNA gene amplicon sequencing, we compared bacterial diversity, taxonomic composition, and community structure among the sampled sites. We also examined patterns when samples were grouped according to the phytophysiognomy represented at each site. Given the environmental heterogeneity of the study area, we expected bacterial communities to show site-associated differences in diversity and composition. This study provides a baseline for understanding soil bacterial variation across contrasting natural and managed environments in the southern Peruvian Andes.

## 2. Materials and Methods

### 2.1. Study Area and Historical Aridity Characterization

The study was conducted in the province of Candarave, Tacna region, southern Peru, within the headwaters of the Locumba River basin [13]. The area comprises high-Andean landscapes ranging from approximately 2500 to >4000 m a.s.l. and is characterized by cold mountain conditions, a semi-arid to arid climate, a rainy season concentrated during the austral summer, and a prolonged dry period during the rest of the year [13,14,15]. The dominant land-cover types include herbaceous vegetation, shrublands, succulent vegetation, areas with sparse vegetation, wetlands, *Polylepis* woodlands, and agricultural lands [16].

Aridity was estimated from the relationship between annual precipitation and potential evapotranspiration using the expression AI = 1 − (P/PET), where *AI* is the annual aridity index, *P* is annual precipitation, and *PET* is annual potential evapotranspiration [17]. Climatic data were obtained from gridded PISCO-SENAMHI datasets (Peruvian interpolated data of the SENAMHI’s Climatological and Hydrological Observations) for the 2000–2016 period, provided as NetCDF (.nc) files at a spatial resolution of 0.1° [18]. This period corresponds to the temporal coverage available for the climatic product used in this study. These data were therefore used as historical climatic descriptors of the sampling sites and do not represent climatic conditions at the time of soil sampling in 2025. Precipitation and PET data were used to calculate annual aridity index values for the grid cells covering the province of Candarave. Temporal variation in aridity was summarized by calculating Pearson’s correlation coefficient between annual AI and year for each grid cell. Pearson’s *r* was used to describe the direction and strength of the linear temporal association, whereas the magnitude of change was quantified separately using the linear trend slope (AI units year^−1^).

To distinguish historical aridity level from temporal change, mean annual AI, Pearson’s correlation coefficient, linear trend slope (AI units year^−1^), *p*-value, and 95% confidence interval of the slope were calculated for each sampling grid cell using the annual AI series from 2000 to 2016. Field soil moisture was measured separately for each sampling replicate in 2025 and represents local soil conditions at the time of sampling rather than long-term climatic aridity.

### 2.2. Sampling Design and Soil Collection

Five sampling sites (P1–P5) were selected along a north–south transect from the upper to the lower zone of Candarave, spanning four grid cells with contrasting Pearson correlation coefficients between annual AI and year. The study considered two complementary analytical perspectives: comparisons among the five sampling sites and exploratory comparisons in which soil samples were grouped according to the phytophysiognomy represented at each site. Soil samples collected within each site were treated as within-site sampling replicates and were processed independently. Site-level comparisons were used to describe spatial variation among P1–P5, whereas phytophysiognomic groupings were used to examine patterns among samples from sites sharing the same vegetation or land-use setting. These analyses were not intended to estimate independent causal effects of vegetation, land use, or historical aridity.

**Figure 1:**
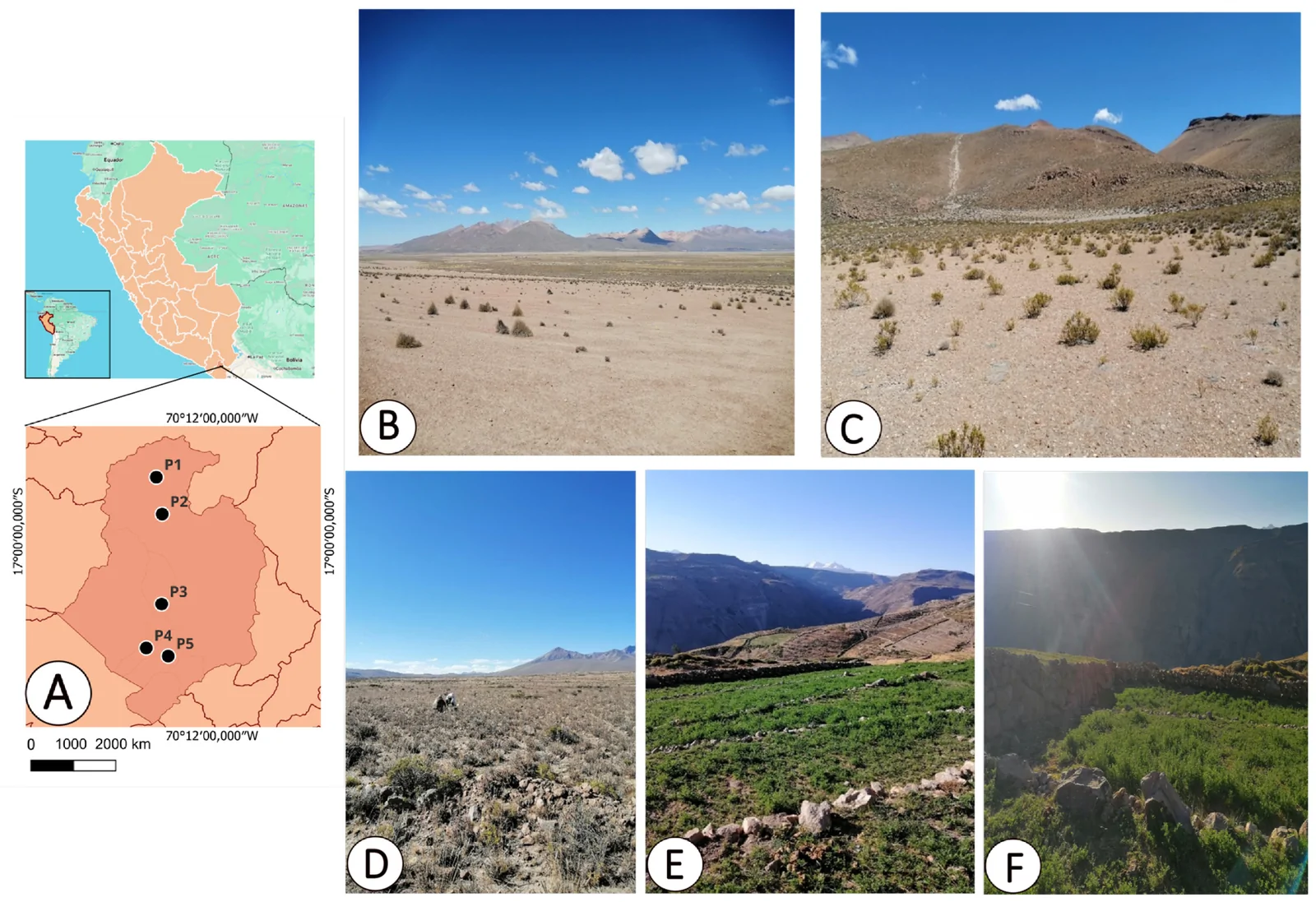
Spatial distribution of the sampling sites in the province of Candarave, Tacna region, southern Peru, generated using QGIS v3.44.7.(**A**) General map of the study area indicating the five sampling points; (**B**,**C**) sampling points P1 and P2, corresponding to herbaceous–shrubland sites; (**D**) sampling point P3, corresponding to the succulent shrubland site; (**E**,**F**) sampling points P4 and P5, corresponding to agricultural sites.

Soil sampling was conducted during the dry season on 16 August 2025. Five within-site sampling replicates were established at each site. After removing surface litter and loose non-soil material, soil was collected from the 0–10 cm layer. Each replicate consisted of 10 subsamples randomly collected within an area of approximately 1 m^2^ and combined and homogenized in the field to yield approximately 250 g of soil [19,20]. Sampling replicates were spatially separated within each site, and their individual coordinates are provided in Supplementary Table S2. Samples were placed in sterile Ziploc bags, transported to the laboratory within 24 h of collection in a cooler with frozen gel packs at approximately 4 °C, and subsequently stored at −20 °C until processing [21]. Of the 25 samples initially collected, P1R5 and P5R1 were excluded prior to DNA extraction because they did not meet the quality requirements for molecular analysis after transport; therefore, 23 samples were processed for DNA extraction, sequencing, and downstream analyses. Prior to DNA extraction, the samples were sieved to remove coarse particles and homogenized, and each sample was processed separately for DNA extraction and sequencing. Sample tracking is summarized in Supplementary Table S1, and the sampling sites were documented using photographic records and field observations presented in Figure 1 and summarized in Table 1.

Volumetric soil water content and soil temperature were measured in situ at each sampling replicate using a WET-2 sensor connected to an HH2 Moisture Meter (Delta-T Devices Ltd., Cambridge, UK). The sensor was inserted directly into the soil within the 0–10 cm sampling layer, and measurements were recorded after approximately 1 min of stabilization. Soil water content was expressed as a percentage (%) and temperature in °C. Soil pH was measured in the field using a portable HI99121 pH meter (Hanna Instruments, Woonsocket, RI, USA) in a soil–distilled water suspension, with readings recorded after approximately 1 min of stabilization. The instruments were checked and calibrated prior to field measurements, and the pH meter was calibrated using standard buffer solutions.

### 2.3. Soil DNA Extraction, 16S rRNA Amplification, and Sequencing

Total genomic DNA was extracted independently from each soil sample using the NucleoSpin Soil Mini kit for DNA from soil (Macherey-Nagel, Düren, Germany), following the manufacturer’s instructions. DNA concentration was quantified using a Qubit 4 Fluorometer (Thermo Fisher Scientific, Waltham, MA, USA), and DNA purity was assessed using an Agilent BioTek Epoch 2 Microplate Spectrophotometer (Agilent, Santa Clara, CA, USA) based on the A260/A280 absorbance ratio. The extracted DNA samples were then sent to Austral-Omics (Valdivia, Chile) for 16S rRNA gene amplicon sequencing. At the sequencing facility, the V3–V4 hypervariable region was amplified using primers 341F (5′-CCTACGGGNGGCWGCAG-3′) and 785R (5′-GACTACHVGGGTATCTAATCC-3′), which provide broad bacterial coverage for 16S rRNA amplicon profiling [22]. Amplicon library preparation, indexing, normalization, and sequencing were performed by Austral-Omics using the Illumina NextSeq 2000 (Illumina, San Diego, CA, USA) platform with a paired-end sequencing configuration of 2 × 300 bp, targeting approximately 100,000 reads per sample.

### 2.4. Bioinformatic Processing and Taxonomic Assignment

Raw sequencing reads were subjected to quality control using BBDuk v38.34 from the BBMap package to remove Illumina adapters, sequencing artifacts, and PhiX Control v3 sequences. Quality filtering was performed using the following parameters: ktrim = l, *k* = 23, mink = 11, hdist = 1, minlen = 50, tpe, tbo, qtrim = rl, trimq = 20, ftm = 5, and maq = 20. The filtered reads were then imported into QIIME2 v2024.10 for downstream bioinformatic analyses following the “Moving Pictures” pipeline [23].

Amplicon sequence variants (ASVs) were inferred using the q2-dada2 plugin in QIIME2, which performs sequence quality filtering, dereplication, paired-end read merging, and chimera removal using default parameters (no truncation applied, truncLen = 0) [24]. The number of reads retained after each quality control step is presented in Supplementary Table S3. A no-template PCR control (NTC), an extraction blank, and a positive control were sequenced together with the soil samples and processed with the same pipeline. The NTC and the extraction blank yielded 282 and 7339 reads, respectively, both below 4% of the median sequencing depth of the samples, whereas the positive control yielded 354,797 reads. Reads in the extraction blank were distributed across many ASVs at low counts (at most 899 reads per ASV) and were mostly assigned to taxa typical of soil, such as *Gemmataceae*, *Isosphaeraceae*, and *Microtrichaceae*, a pattern consistent with low-level cross-talk among samples rather than reagent contamination. Control samples were excluded from all downstream analyses. A total of 58,095 ASVs were inferred across all samples using the q2-dada2 plugin, and no additional frequency- or prevalence-based filtering was applied to the resulting feature table. Taxonomic assignment of ASVs was performed using the q2-feature-classifier plugin with the classify-sklearn method [25]. Classification was conducted against the SILVA 138 Ref NR 99 database, previously trained with a Naive Bayes classifier [26]. ASVs assigned to Archaea, chloroplasts, or mitochondria, or left unassigned (217 ASVs), represented only 0.25% of the total reads, so their presence has a negligible effect on the diversity and compositional patterns reported. Relative sequence proportions represent each taxon’s share of total reads per sample and do not necessarily reflect cellular abundance because amplicon proportions can be influenced by DNA extraction efficiency, primer affinity, amplification efficiency, and variation in 16S rRNA gene copy number.

An exploratory STAMP screening was conducted on non-rarefied data to identify candidate taxa for subsequent site-level evaluation. For this screening, samples were grouped as herbaceous–shrubland, succulent shrubland, and agricultural, and differences among groups were assessed using the Kruskal–Wallis H test in STAMP v2.1.3 [27], with Benjamini–Hochberg correction for multiple comparisons. No prevalence-based filtering was applied before this screening. Taxa meeting both *q* ≤ 0.05 and an eta-squared effect size ≥ 0.90 were retained as candidates. Analyses were performed separately at each taxonomic level, with multiple-testing correction applied independently within 47 phyla, 140 classes, 383 orders, 622 families, and 1275 genera. Because this screening treated within-site samples as replicates of phytophysiognomic groups and did not account for the compositional nature of sequencing data, its results were not interpreted as formal evidence of differences among phytophysiognomies. The identified candidate taxa were subsequently evaluated using ANCOM-BC [28] via the QIIME2 q2-composition plugin, with the sampling site designated as the grouping factor. This method accounts for differences in sampling fractions across the dataset. For the ANCOM-BC analysis, we used the non-rarefied feature table after excluding the control samples and collapsed the data at the genus, family, and order levels. Taxa present in fewer than 10% of the samples were excluded. To perform all pairwise comparisons among sites, we iteratively fitted the model using each site as the reference level. All resulting *p*-values were adjusted using the Holm method within each comparison (*q* < 0.05). Consequently, only taxonomic differences supported by the ANCOM-BC analysis were reported. To descriptively examine shared bacterial families and genera, as well as taxa detected only within individual phytophysiognomic groups, Venn diagrams were generated using the InteractiVenn web tool (https://www.interactivenn.net/; accessed on 21 August 2026) [29]. Presence was defined as the detection of a taxon in the pooled samples of a given phytophysiognomic group, without applying a minimum abundance or prevalence threshold. The herbaceous–shrubland, succulent shrubland, and agricultural groups comprised 9, 5, and 9 samples, respectively, and taxa detected only within one group were defined as those present in the pooled samples of that group but not detected in the other two groups.

### 2.5. Diversity and Statistical Analyses

Microbial diversity and statistical analyses were performed using the QIIME2 v2024.10 platform [23]. Prior to diversity analyses, the feature table was rarefied to a standardized sequencing depth based on the sample with the lowest number of reads (109,982 reads, P3R5) to minimize biases associated with differences in sequencing effort among samples. Sequencing coverage and sampling sufficiency were evaluated using alpha rarefaction curves based on observed features. The phylogenetic tree was constructed using the q2-phylogeny align-to-tree-mafft-fasttree plugin with default parameters. Alpha diversity was estimated using observed features, the Shannon diversity index, Faith’s phylogenetic diversity, and Pielou’s evenness index [30]. Alpha-diversity metrics were compared among the five sampling sites (P1–P5) using the Kruskal–Wallis test (*p* < 0.05), followed by pairwise Kruskal–Wallis comparisons between sites with Benjamini–Hochberg false discovery rate (FDR) correction (*q* < 0.05). Since each phytophysiognomy was represented by a maximum of two sites, the samples within a given site could not serve as independent replicates for these broader vegetation categories. Accordingly, variation among phytophysiognomies was described only as an observational pattern, without formal statistical testing.

Beta diversity was assessed using unweighted UniFrac distances [31] calculated from the rarefied feature table (109,982 sequences per sample). Principal Coordinates Analysis (PCoA) was performed using the q2-diversity PCoA method to assess differences in bacterial community structure among samples, and the resulting ordinations were visualized with Emperor [32]. To visualize the taxa most strongly aligned with the ordination pattern, a PCoA biplot was generated with the q2-diversity PCoA-biplot method using the genus-level rarefied feature table. The 10 features with the largest vector magnitudes were projected onto the ordination space (q2-emperor biplot, number-of-features = 10). These vectors were used descriptively and were not subjected to independent hypothesis testing or multiple-testing correction. Differences in bacterial community composition among the five sampling sites (P1–P5) were assessed using Permutational Multivariate Analysis of Variance (PERMANOVA) [33] with the q2-diversity beta-group-significance method (method = permanova, 999 permutations, *p* < 0.05). Pairwise comparisons among sampling sites were performed using the same approach, with *p*-values corrected for multiple comparisons using the Benjamini–Hochberg FDR [34]. Homogeneity of multivariate dispersion among sampling sites was assessed using Permutational Analysis of Multivariate Dispersion (PERMDISP) [35] with the q2-diversity beta-group-significance method (method = permdisp, 999 permutations, *p* < 0.05).

### 2.6. Functional Prediction Analysis

Functional profiles were predicted using the q2-picrust2 plugin (v2024.5_1) in QIIME2, implementing PICRUSt2 [36], with the representative ASV sequences and ASV feature table as inputs. Hidden State Prediction (HSP) was conducted using the PIC (Phylogenetic Investigation of Communities by Reconstruction of Unobserved States) method. Functional profiles were predicted in terms of MetaCyc metabolic pathways. To ensure prediction reliability, ASVs with a Nearest Sequenced Taxon Index (NSTI) score greater than 2.0 were filtered out. Subsequently, pathway abundances were normalized by predicted 16S rRNA gene copy numbers and inferred using the MetaCyc database [37]. Of the 65 MetaCyc functional pathways predicted by PICRUSt2, we focused on a subset of 17 for downstream analysis. We specifically targeted pathways involved in carbon and nitrogen cycling and organic matter degradation, given their central role in the soil biogeochemical dynamics explored here.

Statistical analyses and data visualizations were performed in R v4.5.3 using the vegan package [38], adapting the analytical scripts provided by Torondel et al. (2016) [39]. Non-metric multidimensional scaling (NMDS) was performed with the metaMDS() function on a Bray–Curtis dissimilarity matrix, using two dimensions (*k* = 2) and up to 1000 random starts (trymax = 1000). Ordination goodness of fit was assessed by the final stress value, and values below 0.05 were considered to indicate an excellent representation of the dissimilarity matrix [40]. To identify the subset of MetaCyc level 3 categories most strongly associated with community dissimilarity, the BIO-ENV procedure was applied using the bio.env() function [41]. For this analysis, both the genus abundance matrix and the MetaCyc category matrix were converted into Bray–Curtis dissimilarities, which were compared using Spearman’s rank correlation. The BVSTEP procedure [42] was then applied with the bv.step() function, using the same distance settings, to identify the subset of genera that best reproduced the overall community pattern. In each of 10 restarts, 30% of the 1266 genera were randomly selected, and the search stopped when the correlation reached 0.95 or increased by less than 0.0001 between steps (random seed = 123). The selected subsets of MetaCyc categories and genera were projected as vectors onto the NMDS ordination using the envfit() function. Because these subsets were selected from the same data, envfit permutation *p*-values are not reported, and the vectors are used only to visualize associations. Arrow directions indicate the direction of increasing values, and arrow lengths are proportional to the strength of the correlation with community structure.

The functional profiles were derived directly from the community ASV table, rendering the two datasets mathematically dependent. Therefore, the BIO-ENV and envfit procedures were employed purely for the exploratory visualization of taxonomic-functional links, without formal inferential intent, and the predicted pathways are treated as hypotheses for future metagenomic or transcriptomic testing.

## 3. Results

### 3.1. Environmental and Edaphic Characterization of Sampling Sites

**Figure 2:**
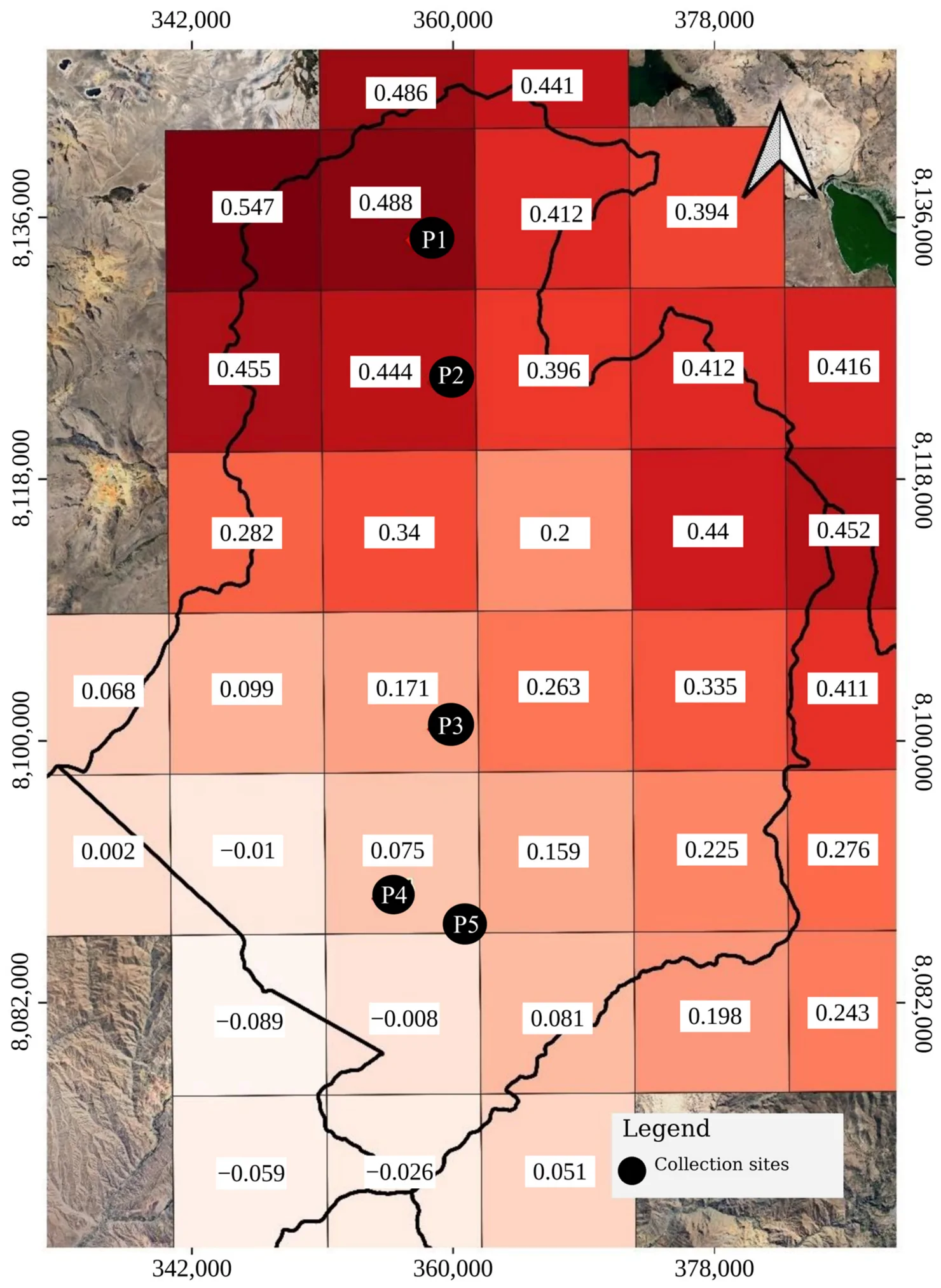
Spatial distribution of Pearson’s correlation coefficients between annual aridity index and year in the province of Candarave, Tacna, Peru, for the 2000–2016 period. Darker colors indicate stronger positive temporal associations between annual AI and year, whereas lighter colors indicate weaker or negative associations.

The sampling sites differed in elevation, soil moisture, soil temperature, pH, and historical aridity descriptors (Table 2, Figure 2, and Supplementary Table S4), whereas vegetation cover and land-use characteristics are summarized in Table 1. P1 and P2 were located at the highest elevations, approximately 4490–4532 m a.s.l., and corresponded to herbaceous–shrubland vegetation. These sites showed the strongest positive temporal associations between annual AI and year among the sampled sites (Pearson’s *r* = 0.488 and 0.444, respectively), with corresponding linear trend slopes of 0.0257 and 0.0185 AI units year^−1^. Soil pH was predominantly acidic, and field soil moisture ranged from 8.9% to 20.9% at P1 and from 5.0% to 11.7% at P2.

P3, corresponding to succulent shrubland, showed a weaker positive temporal association between annual AI and year (*r* = 0.171; slope = 0.0039 AI units year^−1^) and was located at approximately 4013–4020 m a.s.l. Field soil moisture was very low at this site, ranging from 0.2% to 1.9% in four replicates, with one replicate below the detection range of the equipment. P4 and P5 were located at lower elevations and corresponded to agricultural areas. Both sites showed the weakest positive temporal association among the sampled sites (*r* = 0.075; slope = 0.0015 AI units year^−1^) and had soil pH values ranging from near neutral to slightly alkaline.

The spatial distribution of Pearson’s correlation coefficients between annual AI and year for the 2000–2016 period is shown in Figure 2, whereas mean annual AI, Pearson’s *r*, linear trend slope, and field soil moisture are reported separately in Supplementary Table S4. Mean annual AI values were 0.220, 0.406, 0.775, 0.843, and 0.843 for P1–P5, respectively, while the corresponding Pearson correlation coefficients were 0.488, 0.444, 0.171, 0.075, and 0.075. Thus, the highest mean annual AI values occurred at P4 and P5, whereas the strongest positive temporal associations occurred at P1 and P2. Field soil moisture showed a different pattern, particularly at P3. These descriptors were therefore considered separately throughout the study.

### 3.2. Taxonomic Structure and Composition of Soil Bacterial Communities

**Figure 3:**
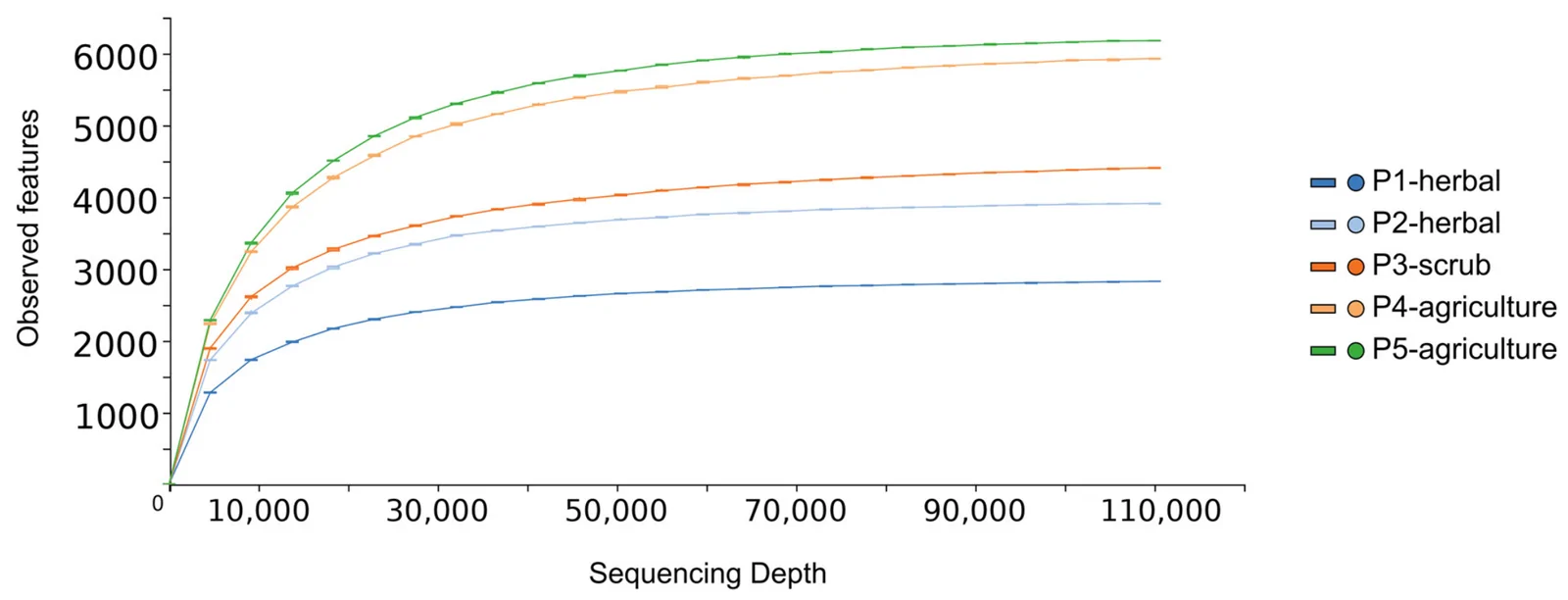
Alpha rarefaction curves based on observed features for soil samples collected across the five sampling sites in Candarave, Tacna, Peru. Each curve represents the number of observed features for replicates from each sampling site (P1, *n* = 4; P2, *n* = 5; P3, *n* = 5; P4, *n* = 5; P5, *n* = 4) at each sequencing depth, up to the rarefaction depth of 109,982 sequences per sample.

Bioinformatic processing yielded 5,277,163 high-quality sequences across the dataset, with an average of 229,442 reads per sample (Supplementary Table S3), and produced 58,095 ASVs. Read-retention rates varied among samples, with the largest reductions occurring during paired-end read merging, particularly in several samples from P3 and in P4R2 (Supplementary Table S3). Bacterial taxa dominated the dataset, whereas 217 ASVs (0.37%) corresponded to Archaea (123), chloroplasts (64), mitochondria (25), or remained unassigned (5). These features were retained in the matrix used for diversity and functional profiling. Given the bacterial focus of this study, archaeal detections were not interpreted further. Alpha rarefaction curves approached a plateau at the selected sequencing depth, indicating approximate saturation of the observed features rather than complete capture of bacterial richness (Figure 3).

**Figure 4:**
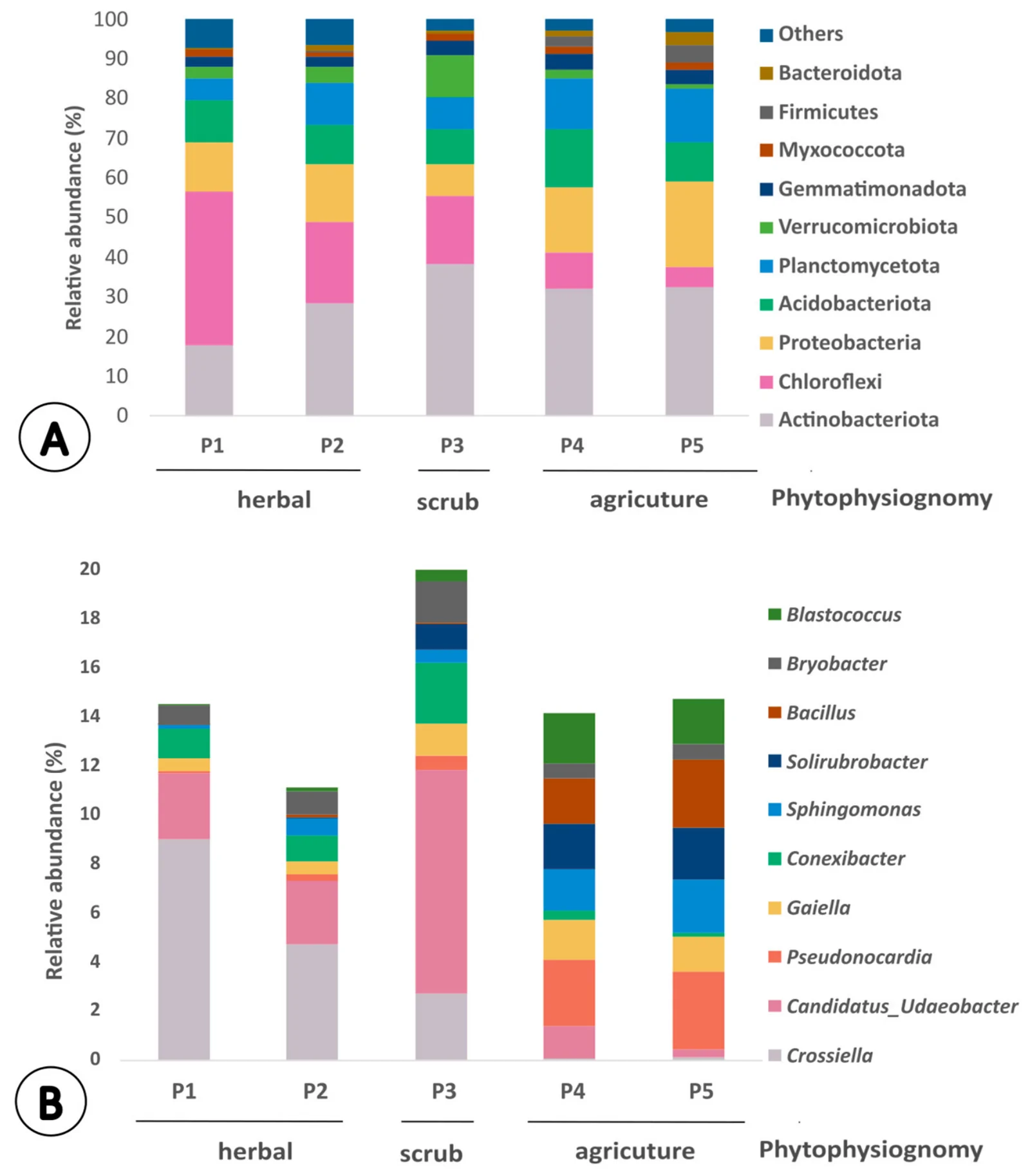
Relative sequence proportions of dominant bacterial taxa across sampling sites. (**A**) Phylum level; (**B**) genus level, showing the 10 genera with the highest relative sequence proportions. Bars represent the mean relative sequence proportion across replicates at each site.

The taxonomic profiles were dominated by bacteria. At the phylum level, *Actinobacteriota*, *Chloroflexota*, and *Proteobacteria* represented the major bacterial groups across the five sampling sites, but their relative sequence proportions varied among sites (Figure 4A). *Chloroflexota* showed the highest proportion in P1, whereas *Actinobacteriota* was the dominant phylum in P2, P3, P4, and P5. *Proteobacteria* showed comparatively higher proportions in P4 and P5, where it exceeded *Chloroflexota*.

At the genus level, the proportion of taxonomically identified sequences varied among sampling sites. The highest proportion of sequences assigned at the genus level was observed in P5 (44.34%), followed by P4 (34.42%), whereas lower proportions were recorded in P2 (23.94%), P3 (21.48%), and P1 (17.92%). Conversely, sequences unresolved at the genus level represented a large fraction of the bacterial community across all sites, ranging from 56.14% in P5 to 82.07% in P1. This pattern indicates that a substantial fraction of the bacterial community in these high-Andean soils remained unresolved at the genus level.

Marked differences in genus-level composition were observed among sampling sites (Figure 4B). P1 was characterized by a high relative sequence proportion of *Crossiella*, which was also the most represented genus shown for P2, although at a lower proportion. In P3, *Candidatus Udaeobacter* showed the highest relative sequence proportion, followed by *Crossiella* and *Conexibacter*. In P4 and P5, the genus-level profiles were more evenly distributed among several dominant taxa, particularly *Pseudonocardia*, *Bacillus*, *Sphingomonas*, *Solirubrobacter*, and *Blastococcus*.

**Figure 5:**
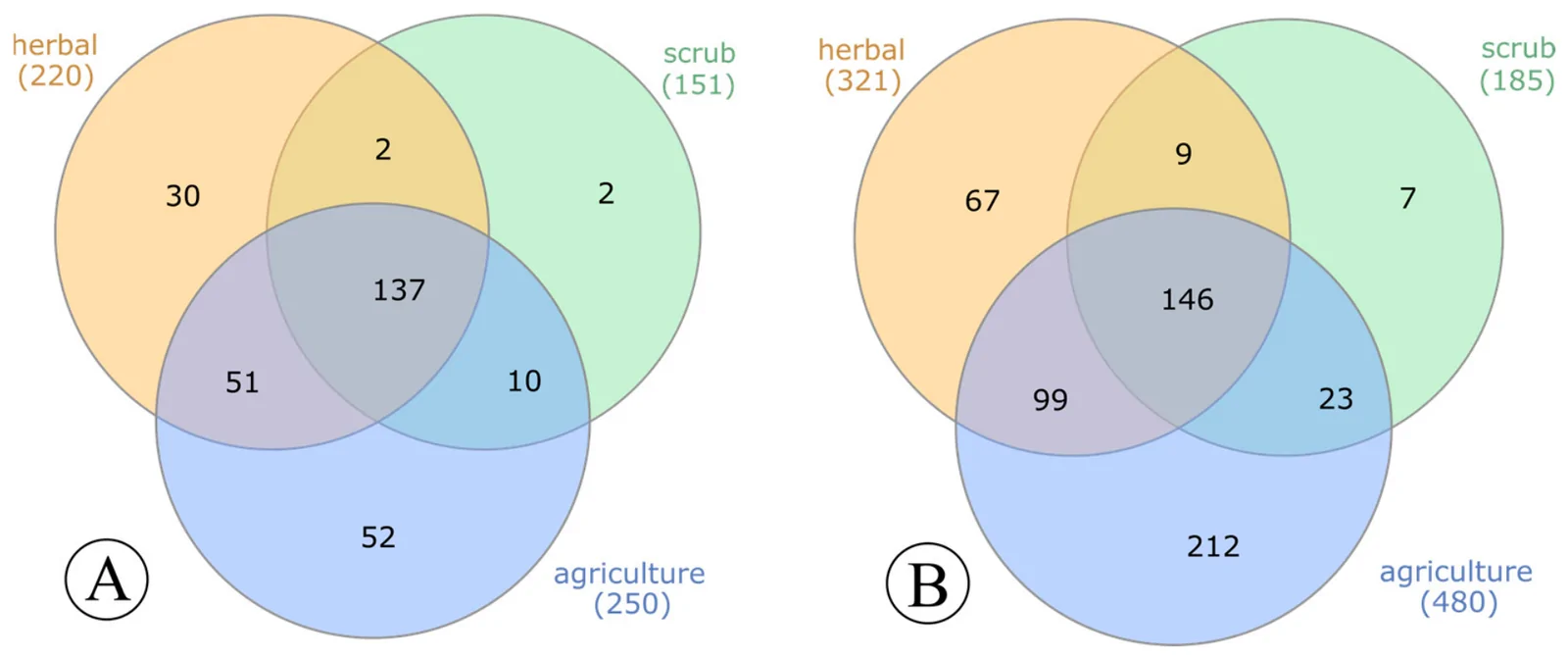
Distribution of bacterial families (**A**) and genera (**B**) detected across the pooled herbaceous–shrubland, succulent shrubland, and agricultural groups, including taxa detected in all groups and taxa detected only within individual groups.

The Venn diagrams provided a descriptive comparison of bacterial families and genera detected when samples were pooled according to phytophysiognomy (Figure 5). A total of 137 families and 146 genera were detected in all three pooled categories. At the family level, 52 families were detected only in the agricultural category, 30 only in herbaceous–shrubland, and two only in succulent shrubland. At the genus level, the corresponding numbers were 212, 67, and seven, respectively. Among genera shared by exactly two categories, the largest overlap occurred between herbaceous–shrubland and agricultural samples (99 genera), followed by succulent shrubland and agricultural samples (23 genera), whereas herbaceous–shrubland and succulent shrubland shared nine genera. These patterns are descriptive because taxon presence was determined from pooled samples without a minimum prevalence threshold, and the categories differed in sample number and sequencing depth. The complete list of genera detected only within each category is provided in Supplementary Table S5.

Differential abundance among sampling sites was assessed using ANCOM-BC. At the genus level, the number of genera differing significantly between pairs of sites (*q* < 0.05) was lowest between sites sharing the same phytophysiognomy (50 genera between P4 and P5 and 92 between P1 and P2) and highest between P3 and P4 (378 genera) and between P2 and P4 (288 genera). The nine taxa identified in the exploratory STAMP screening were evaluated with ANCOM-BC at their respective taxonomic ranks, and site-level differences were supported for all nine taxa, although the specific site contrasts differed (Supplementary Figure S1). *Azospirillales* was more abundant at both agricultural sites (P4 and P5) than at P1, P2, and P3. *Geminicoccaceae*, *Blastococcus*, and *Solirubrobacter* were more abundant at P4 than at P1, P2, and P3, whereas at P5, *Blastococcus* was more abundant than at P2 only, and *Geminicoccaceae* and *Solirubrobacter* did not differ from the other sites. *Nitriliruptoraceae* and *Kineosporiaceae* were more abundant at P3 than at all other sites, and Elsterales, *Candidatus Udaeobacter*, and *Conexibacter* were more abundant at P3 than at P2, P4, and P5. *Elsterales* and *Conexibacter* were also less abundant at the agricultural sites than at P2 and P3.

The taxa more abundant at the agricultural sites were affiliated with *Actinobacteriota* and *Proteobacteria*, whereas those more abundant at P3 included members of Verrucomicrobiota, *Actinobacteriota*, and *Proteobacteria*.

### 3.3. Alpha and Beta Diversity and Community Structuring

Alpha-diversity metrics differed among the five sampling sites (Table 3 and Supplementary Figure S2). Observed ASV richness differed among sites (Kruskal–Wallis, H = 15.33, *p* = 0.004), with the lowest values at P1, which differed from all other sites. P2 also showed lower richness than the agricultural sites (P4 and P5), whereas P3 showed intermediate values and did not differ significantly from P2, P4, or P5.

Shannon diversity also differed among the five sampling sites (H = 17.67, *p* = 0.001), with the lowest values at P1 and P2, intermediate values at P3, and the highest values at P4 and P5, which differed from all other sites. Faith’s phylogenetic diversity differed among sites (H = 13.40, *p* = 0.009), with lower values at P1 than at P2, P3, and P4, while no significant differences were detected among the remaining sites. Pielou’s evenness also differed among sites (H = 16.81, *p* = 0.002), with the highest values at P4 and P5. Evenness at P2 was lower than at P3, P4, and P5, whereas P1 showed the lowest mean value but high variability among samples. Overall, P4 and P5 showed the highest values across the diversity metrics and did not differ significantly from each other, whereas P1 and P2 generally showed lower values.

Beta diversity analyses revealed marked variation in bacterial community composition among the five sampling sites. Principal Coordinates Analysis (PCoA) based on unweighted UniFrac distances showed site-associated separation of the samples, with P1 and P2 positioned relatively close to each other, P4 and P5 occupying another region of the ordination space, and P3 showing a distinct intermediate position (Supplementary Figure S3). The first three axes explained 47.29% of the total variation. Site-level PERMANOVA confirmed significant differences in community composition among the five sites (pseudo-F = 4.670, *p* = 0.001, *n* = 23), and all pairwise site comparisons remained significant after Benjamini–Hochberg correction (Supplementary Table S6). Multivariate dispersion did not differ significantly among sites (PERMDISP, F = 0.686, *p* = 0.153; Supplementary Table S7). The significant differences between P1 and P2 and between P4 and P5 also indicate substantial site-to-site heterogeneity within the herbaceous–shrubland and agricultural groupings, respectively.

The taxon vectors projected onto the unweighted UniFrac PCoA showed the bacterial taxa with the largest vector magnitudes in the ordination space (Supplementary Figure S3). These included *Actinobacteriota* such as *Pseudarthrobacter*, *Crossiella*, and uncultured members of Pseudonocardiaceae, as well as AD3 lineages within *Chloroflexota* and *Proteobacteria* affiliated with Rhizobiales/Beijerinckiaceae and Burkholderiales/Alcaligenaceae. Vector direction indicates the ordination gradient along which the relative sequence proportion of each taxon increased, whereas vector length reflects the strength of its association with the ordination pattern. These relationships are descriptive features of the ordination and do not represent independent tests of ecological causation.

**Figure 6:**
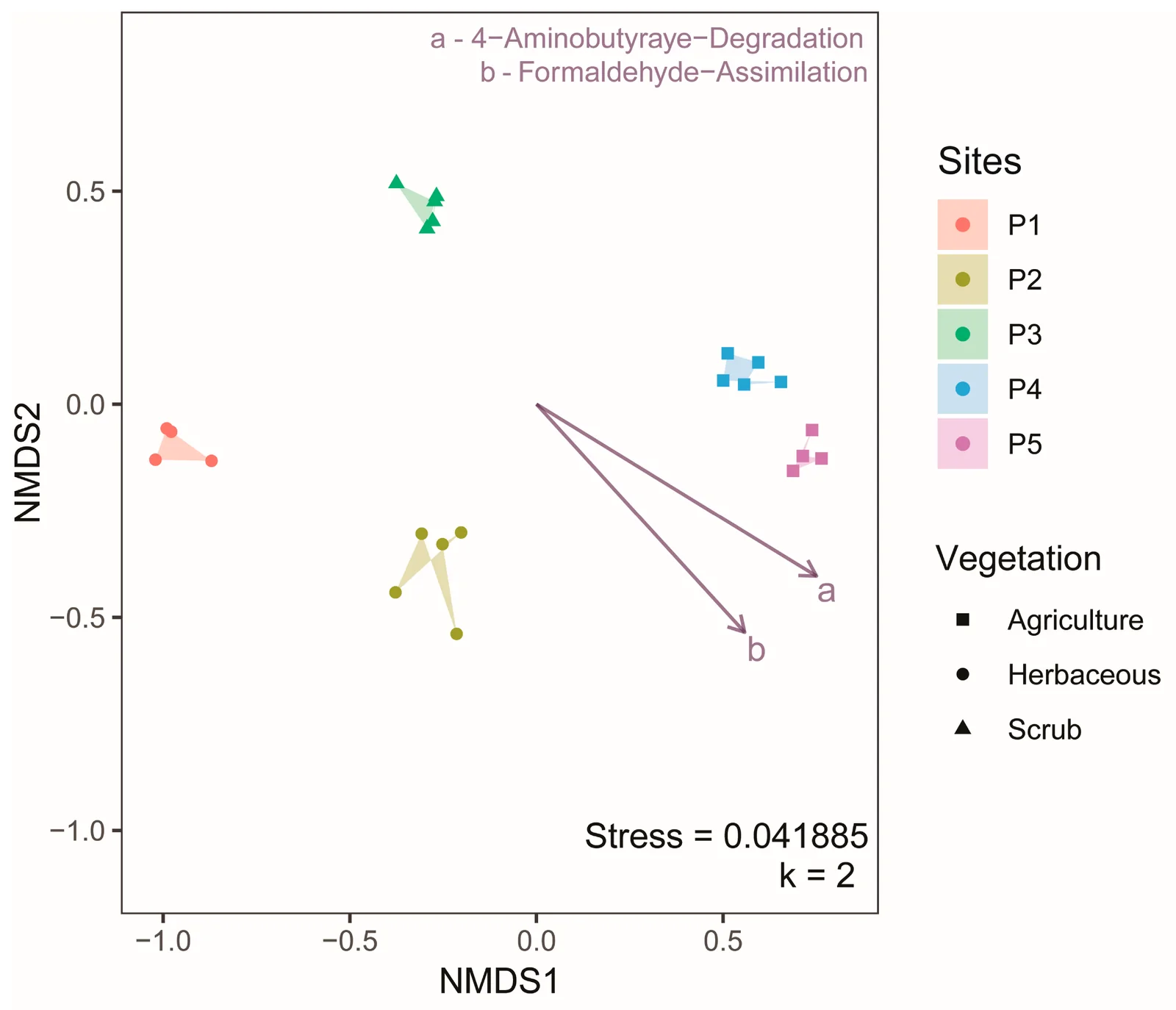
Non-metric multidimensional scaling (NMDS) ordination of soil bacterial genus-level relative sequence profiles across sampling sites based on Bray–Curtis dissimilarity (stress = 0.042; *k* = 2). Arrows represent the MetaCyc functional categories selected by the BIO-ENV procedure from 17 candidate categories as the subset most closely associated with community dissimilarity (Spearman’s ρ = 0.354): (a) 4-aminobutyrate degradation (r^2^ = 0.45) and (b) formaldehyde assimilation (r^2^ = 0.37). Vectors were projected onto the ordination using envfit for exploratory visualization. Arrow direction indicates the gradient along which each predicted category increases, and arrow length reflects its association with the ordination pattern.

NMDS ordination based on Bray–Curtis dissimilarities of genus-level profiles showed distinct clustering among the five sampling sites, with a low stress value (stress = 0.042; *k* = 2), indicating a good representation of community dissimilarities in two dimensions (Figure 6). Among the 17 predicted MetaCyc pathways evaluated, the BIO-ENV procedure selected 4-aminobutyrate degradation and formaldehyde assimilation as the subset most closely associated with community dissimilarity (Spearman’s ρ = 0.354). In the exploratory envfit analysis, 4-aminobutyrate degradation (r^2^ = 0.45) and formaldehyde assimilation (r^2^ = 0.37) were oriented toward P4 and P5. These associations are descriptive because the pathways were selected and evaluated using the same dataset and represent predicted functional potential rather than direct evidence of metabolic activity.

## 4. Discussion

Soil bacterial communities differed markedly among the five high-Andean sites of Candarave. Differences were evident in taxonomic composition, alpha diversity, and beta diversity across the five sampling sites. Because historical aridity descriptors, local soil conditions, elevation, vegetation, and land use varied concurrently across the study area, these patterns reflect substantial site-associated heterogeneity within a complex environmental setting. Similar studies in drylands have reported associations between soil bacterial community structure and variation in water availability, edaphic conditions, vegetation, and land-use history [4,8,43].

At the phylum level, *Actinobacteriota*, *Chloroflexota*, and *Proteobacteria* were the dominant bacterial groups across the five sites, although their relative sequence proportions varied among them. *Chloroflexota* was more strongly represented at P1, whereas *Actinobacteriota* predominated at P2–P5. *Proteobacteria* showed comparatively higher proportions at P4 and P5. The high relative sequence proportion of *Actinobacteriota* is consistent with reports from high-altitude and arid soils of the Central Andes, where members of this phylum are frequently detected and include lineages adapted to desiccation, nutrient limitation, and high radiation [5,6,44].

Alpha-diversity patterns did not align consistently with the historical aridity descriptors across the five sites. At the site level, P4 and P5 generally showed the highest richness, Shannon diversity, phylogenetic diversity, and evenness values, whereas P1 and P2 showed lower values and P3 occupied an intermediate position for most metrics. These differences cannot be assigned to a single environmental factor. P4 and P5 had the highest mean annual aridity index values but the weakest historical aridity-trend estimates, whereas P3 had very low field soil moisture. The agricultural sites also occurred at lower elevations and had near-neutral to slightly alkaline soils, while P1 and P2 were located at higher elevations and had predominantly acidic pH. The observed alpha-diversity pattern therefore occurred within a broader set of co-varying environmental differences. Broad dryland studies have often reported declining microbial diversity with increasing aridity, although local soil properties and land-use context can modify these relationships [2,3,45].

The high-elevation sites P1 and P2 also showed distinctive genus-level profiles. P1 had the largest proportion of sequences unresolved at the genus level and a comparatively high representation of *Crossiella*, which was also among the most represented genera at P2. High proportions of taxonomically unresolved bacteria have been reported in other poorly characterized high-Andean and Atacama soils, where environmental sequencing has revealed substantial diversity that remains underrepresented in reference databases [6,46,47]. *Crossiella* is a relatively uncommon actinobacterial genus reported from diverse environmental habitats, including oligotrophic and mineral-associated environments [48]. Its higher relative sequence proportion at P1 contributes to the distinctive taxonomic profile of this site, although the available data do not allow this pattern to be associated specifically with elevation, acidic pH, vegetation, or the historical aridity-trend estimate.

P3 combined an intermediate historical aridity-trend estimate with very low field soil moisture (0.2–1.9%; one replicate below detection), showing that these descriptors reflect environmental conditions at different temporal scales. Its bacterial community showed high relative sequence proportions of *Candidatus Udaeobacter* and comparatively greater proportions of *Conexibacter*, *Nitriliruptoraceae*, *Kineosporiaceae*, and *Elsterales*. *Candidatus Udaeobacter* is a widespread soil lineage with a reduced genome and metabolic traits associated with persistence under resource-limited conditions [49,50]. Its prominence at P3 is noteworthy, but the concurrent variation in soil moisture, vegetation, and historical aridity prevents linking this pattern to any single environmental condition.

Azospirillales was more abundant at both agricultural sites (P4 and P5), whereas *Blastococcus*, *Solirubrobacter*, and *Geminicoccaceae* were more abundant mainly at P4. *Blastococcus* has been associated with substrate utilization, nutrient transport, and tolerance to environmental stress [51], whereas *Solirubrobacter* is widely distributed in soils and rhizospheres [52]. Some members of *Geminicoccaceae* have also been reported from desert soils and exhibit traits associated with desiccation and other environmental stresses [53]. Agricultural management can modify soil bacterial communities, although the magnitude and direction of these changes vary among systems [9,54]. In Candarave, P4 and P5 occurred at lower elevations and had near-neutral to slightly alkaline soils, but their bacterial communities still differed significantly from each other. The taxonomic patterns observed at these sites therefore reflect their broader site-specific environmental context rather than a uniform agricultural signature.

Community-level analyses further supported strong site-specific heterogeneity. Unweighted UniFrac PCoA and site-level PERMANOVA showed significant differences among all five sites, including P1 versus P2 and P4 versus P5, while multivariate dispersion did not differ significantly among sites. Thus, the relative proximity of some sites in the ordination did not indicate similar bacterial community composition within the same phytophysiognomic group. Comparable spatial variation has been reported in dryland soils, where vegetation, edaphic conditions, and land-use history are associated with differences in bacterial community composition [4,8,9]. The Venn analysis provided a complementary descriptive view, with 137 families and 146 genera detected in all three pooled groups, together with taxa detected only in individual groups. Because these comparisons were based on pooled presence without a minimum prevalence threshold, they describe patterns of taxon detection rather than a defined core microbiome or habitat-specific assemblages.

Predicted functional profiles provided an exploratory view of the taxonomic differences among sites. Among the 17 MetaCyc pathways evaluated, 4-aminobutyrate degradation and formaldehyde assimilation showed the strongest correspondence with community dissimilarity and were oriented toward P4 and P5 in the NMDS ordination. The 4-aminobutyrate metabolism is linked to glutamate metabolism and bacterial stress responses [55], whereas formaldehyde assimilation is involved in C1 metabolism and carbon processing in methylotrophic microorganisms [56]. These associations are consistent with differences in predicted metabolic potential among sites, with both pathways oriented toward P4 and P5 in the ordination. Because the functional profiles were inferred from the same 16S rRNA gene dataset used to characterize community structure, these relationships are exploratory and do not provide independent evidence of pathway activity.

Several limitations constrain the scope of this study. Phytophysiognomic replication was limited (two herbaceous–shrubland sites, one succulent shrubland site, and two agricultural sites), while historical aridity, elevation, soil moisture, pH, vegetation, and land use varied concurrently among sites, preventing their individual contributions from being separated. Although both the no-template control and the extraction blank yielded relatively few reads, only one control of each type was sequenced, and no specific decontamination procedures or filtering based on sequence frequency or prevalence were applied. Furthermore, read retention rates were site-associated, mainly because of lower read-merging efficiency at P3, so technical and site effects cannot be fully separated. In addition, reads assigned to archaeal or organellar ASVs or left unassigned accounted for 0.25% of the total reads and remained in the final dataset. These methodological constraints warrant careful interpretation of downstream analyses that are highly sensitive to rare taxa, most notably unweighted UniFrac distances and pooled presence–absence comparisons. Relative sequence data are also compositional, and predicted functional profiles were derived from the same ASV table used for taxonomic analyses, so these patterns are not independent. Broader spatial replication, replicated negative controls, and direct measurements of soil properties and microbial functions will be needed to test these relationships.

Overall, bacterial communities varied markedly among the five high-Andean sites of Candarave in taxonomic composition, diversity, community structure, and predicted functional profiles. These differences occurred across sites that also varied in soil conditions, elevation, vegetation, land use, and historical aridity descriptors. The study provides a regional baseline for soil bacterial diversity in an understudied sector of the southern Peruvian Andes and identifies site-associated patterns that can be examined further with broader spatial replication and more detailed environmental measurements.

## 5. Conclusions

This study provides a descriptive baseline for soil bacterial diversity in an understudied region of the southern Peruvian Andes. The five sampling sites differed markedly in taxonomic composition, alpha-diversity, and community structure. Because soil conditions, elevation, vegetation, land use, and historical aridity descriptors varied concurrently among sites, the observed patterns cannot be attributed to any single factor. Overall, the study documents substantial site-level bacterial heterogeneity in Candarave and expands the microbiological characterization of high-Andean soils in southern Peru.

## Figures and Tables

**Table 1:** Vegetation and land-use characteristics of the five sampling sites in Candarave, southern Peru.

Sampling Site	Vegetation and Land-Use Characteristics
P1	Herbaceous–shrubland vegetation (pajonal–tolar), corresponding to a natural high-Andean site.
P2	Herbaceous–shrubland vegetation (pajonal–tolar), with vegetation cover similar to that observed at P1.
P3	Succulent shrubland vegetation, characterized by the presence of succulent plants and exposed rocky soil surfaces.
P4	Agricultural area, with alfalfa and maize cultivation observed during field sampling.
P5	Agricultural area, also characterized by cultivated soils with alfalfa and maize present during field sampling.

**Table 2:** Environmental and edaphic characteristics of the five sampling sites.

Sampling Site	Sample ID	Altitude (m a.s.l.)	Volumetric Soil Water Content (%)	Soil Temperature (°C)	Soil pH	Pearson’s r (AI vs. Year)
P1	P1R1	4490	8.9	6.8	5.09	0.488
	P1R2	4498	8.9	6.8	6.48	
	P1R3	4498	10.9	12.5	5.85	
	P1R4	4500	20.9	8.4	6.24	
P2	P2R1	4532	5	11.1	5.18	0.444
	P2R2	4530	5.9	12.6	5.67	
	P2R3	4528	5.1	20.8	5.83	
	P2R4	4531	11.7	14.8	5.73	
	P2R5	4528	7.7	14.2	5.82	
P3	P3R1	4020	0.2	24.2	5.83	0.171
	P3R2	4019	0.8	21.9	6.03	
	P3R3	4017	1.9	21.4	6.41	
	P3R4	4014	1.7	23.4	5.88	
	P3R5	4013	Below detection range	23	6.07	
P4	P4R1	3517	10.2	24.8	8.26	0.075
	P4R2	3514	10.2	23.4	7.67	
	P4R3	3508	25.3	23.1	7.41	
	P4R4	3504	18.9	22.2	7.01	
	P4R5	3505	24.9	20.6	6.83	
P5	P5R2	3397	7.4	13.8	7.28	0.075
	P5R3	3396	12.6	14.4	7.75	
	P5R4	3396	19.4	12.2	7.85	
	P5R5	3396	35.2	12.3	7.49	

Note: Pearson’s *r* describes the direction and strength of the linear association between annual aridity index (AI) and year during the 2000–2016 period; it does not represent the magnitude of temporal change or current aridity conditions. Field soil moisture was measured independently in 2025 for each sampling replicate. Five soil samples were initially collected at each sampling site; however, P1R5 and P5R1 were excluded before DNA extraction because they did not meet the sample-quality conditions required for molecular analysis after transport. Therefore, 23 samples were included in the downstream analyses.

**Table 3:** Alpha diversity indices (mean ± SD) for soil samples at each sampling site. Means within the same column followed by different letters differ significantly (pairwise Kruskal–Wallis test with Benjamini–Hochberg correction, *q* < 0.05). Diversity metrics were calculated using feature tables rarefied to 109,982 reads per sample. Phytophysiognomy is shown for descriptive context only.

Site	Phytophysiognomy	*n*	Observed Features	Shannon	Faith’s PD	Pielou’s Evenness
P1	Herbaceous–shrubland	4	2859 ± 528 a	9.52 ± 0.75 a	167.81 ± 21.35 a	0.830 ± 0.050 abc
P2	Herbaceous–shrubland	5	4029 ± 466 b	10.47 ± 0.17 ab	257.92 ± 25.00 b	0.874 ± 0.012 a
P3	Succulent shrubland	5	4307 ± 738 bc	10.79 ± 0.25 b	260.63 ± 15.85 b	0.895 ± 0.003 b
P4	Agricultural	5	5789 ± 1319 c	11.33 ± 0.34 c	297.70 ± 40.90 b	0.909 ± 0.005 d
P5	Agricultural	4	5855 ± 1079 c	11.35 ± 0.17 c	293.03 ± 30.12 ab	0.909 ± 0.011 cd
Kruskal–Wallis			H = 15.33; *p* = 0.004	H = 17.67; *p* = 0.001	H = 13.40; *p* = 0.009	H = 16.81; *p* = 0.002

## Data Availability

The raw 16S rRNA gene amplicon sequencing data generated in this study have been deposited in the NCBI Sequence Read Archive (SRA). The corresponding BioSample accession numbers are SAMN62945125–SAMN62945147. Additional data supporting the findings of this study are included in the article and its Supplementary Materials.

## Supplementary Materials

The following supporting information can be downloaded at https://www.mdpi.com/article/10.3390/microorganisms14102356/s1; Supplementary Table S1. Sample tracking summary from field collection to downstream analyses; Supplementary Table S2. Universal Transverse Mercator (UTM) coordinates and sampling status of individual soil samples collected at the five sampling sites in Candarave, southern Peru; Supplementary Table S3. Number of sequencing reads retained after quality control; Supplementary Table S4. Historical aridity descriptors (2000–2016) and field soil moisture at the five sampling sites; Supplementary Table S5. Bacterial genera detected only in the pooled samples of each phytophysiognomic group; Supplementary Figure S1. Differential abundance of bacterial taxa between pairs of sampling sites, estimated with ANCOM-BC at the order, family, and genus levels. The taxa shown are those identified in the exploratory STAMP screening. Solirubrobacter 1 and 2 correspond to two lineages assigned to this genus in SILVA 138. Values represent log-fold changes for the first site relative to the second, and positive values (red) indicate higher abundance at the first site. Asterisks indicate significant differences (Holm-adjusted *q* < 0.05). P1 and P2, herbaceous–shrubland; P3, succulent shrubland; P4 and P5, agricultural; Supplementary Figure S2. Alpha diversity of soil bacterial communities at each sampling site. (A) Shannon diversity, (B) observed features, (C) Pielou’s evenness, and (D) Faith’s phylogenetic diversity. P1 and P2, herbaceous–shrubland; P3, succulent shrubland; P4 and P5, agricultural. Statistical comparisons among sites are presented in Table 3; Supplementary Figure S3. Principal Coordinates Analysis (PCoA) based on unweighted UniFrac distances showing variation in soil bacterial community composition across the five sampling sites. Arrows represent the 10 bacterial taxa with the largest vector magnitudes in the ordination space. Arrow direction indicates the ordination gradient along which the relative sequence proportion of each taxon increases, whereas arrow length reflects the vector magnitude; Supplementary Table S6. Global and pairwise PERMANOVA results based on unweighted UniFrac distances (999 permutations); Supplementary Table S7. Global and pairwise PERMDISP results based on unweighted UniFrac distances (999 permutations).
