# Supplementary material for: Soil Bacterial Diversity and Community Composition Across Contrasting High-Andean Sites in Candarave, Southern Peru
Source: Microorganisms. 2026 Oct 15;14(10):2356. doi: 10.3390/microorganisms14102356 (PMC13707568; doi:10.3390/microorganisms14102356)
Supplement: Supplementary file 1 [file microorganisms-14-02356-s001.zip › microorganisms-4577416-supplementary.pdf]

Supplementary Materials

Supplementary Table S1. Sample tracking summary from field collection to downstream analyses.

Workflow step	Number of samples	Samples excluded	Description / reason
Field collection	25	–	Five biological replicates were initially collected from each of the five sampling sites (P1–P5).
Pre-DNA extraction quality check	23	P1R5, P5R1	Two samples were excluded prior to DNA extraction because they did not meet the quality requirements for molecular analysis after transport.
DNA extraction	23	–	The remaining samples were processed independently for environmental DNA extraction.
16S rRNA gene amplification and sequencing	23	–	The V3–V4 region was amplified from all DNA extracts, and the resulting amplicons were sequenced on the Illumina NextSeq 2000 platform.
Bioinformatic processing and quality filtering	23	–	All sequenced samples were retained after quality filtering and downstream bioinformatic processing.
Diversity, taxonomic, and functional analyses	23	–	The 23 retained samples were included in the downstream analyses reported in the manuscript.

Note: After bioinformatic processing, a total of 5,277,163 sequences were retained across the 23 analyzed samples, with an average of 229,442 reads per sample.

Supplementary Table S2. Universal Transverse Mercator (UTM) coordinates and sampling status of individual soil samples collected at the five sampling sites in Candarave, southern Peru.

Sampling site	Sample ID	Environment	UTM E (m)	UTM N (m)	Altitude (m a.s.l.)	Sample status
P1	P1R1	Herbaceous–shrubland	358009	8134416	4490	Included
	P1R2		358011	8134436	4498	Included
	P1R3		357989	8134412	4498	Included
	P1R4		358008	8134396	4500	Included
	P1R5		358027	8134410	4504	Excluded
P2	P2R1	Herbaceous–shrubland	359576	8124667	4532	Included
	P2R2		359580	8124693	4530	Included
	P2R3		359556	8124672	4528	Included
	P2R4		359580	8124649	4531	Included
	P2R5		359599	8124675	4528	Included
P3	P3R1	Succulent shrubland	359606	8100796	4020	Included
	P3R2		359600	8100817	4019	Included
	P3R3		359585	8100796	4017	Included
	P3R4		359602	8100779	4014	Included
	P3R5		359627	8100798	4013	Included
P4	P4R1	Agricultural	355767	8089152	3517	Included
	P4R2		355777	8089160	3514	Included
	P4R3		355762	8089153	3508	Included
	P4R4		355757	8089157	3504	Included
	P4R5		355775	8089161	3505	Included
P5	P5R1	Agricultural	361358	8087048	3397	Excluded
	P5R2		361356	8087044	3397	Included
	P5R3		361360	8087052	3396	Included
	P5R4		361358	8087051	3396	Included
	P5R5		361364	8087054	3396	Included

Note: E, easting; N, northing. Coordinates are reported in meters using the Universal Transverse Mercator (UTM) coordinate system, as recorded during field sampling. Samples P1R5 and P5R1 were excluded prior to DNA extraction and were not included in the microbiome analyses.

Supplementary Table S3. Number of sequencing reads retained after quality control.

Sample ID	Raw sequences	Sequences after BBDuk	Sequences after denoising in QIIME2	Sequences after merging in QIIME2	Sequences after chimera removal in QIIME2	Sequences retained after quality control (%)
P1R1	349159	345490	338723	299517	283174	81.10
P1R2	269451	267195	263214	235656	227066	84.27
P1R3	355110	351566	341843	291691	278327	78.38
P1R4	351237	348142	340938	296296	282943	80.56
P1R5*	-	-	-	-	-	-
P2R1	336041	331828	322305	276370	266346	79.26
P2R2	229352	226717	217577	183100	176564	76.98
P2R3	361855	357039	347017	291379	278840	77.06
P2R4	193216	191080	181394	146900	141860	73.42
P2R5	307156	303653	282536	190235	178141	58.00
P3R1	490330	485150	450966	144634	127620	26.03
P3R2	381392	376058	349594	230175	208305	54.62
P3R3	505864	500062	462372	180320	154954	30.63
P3R4	427984	422010	398188	256876	233358	54.52
P3R5	425802	421443	388072	124921	109982	25.83
P4R1	412015	407846	378251	224497	207291	50.31
P4R2	385167	381972	342946	123181	111620	28.98
P4R3	444602	438730	427438	347967	331358	74.53
P4R4	548910	542232	522573	410717	386102	70.34
P4R5	332949	330062	321396	268910	258081	77.51
P5R1*	-	-	-	-	-	-
P5R2	449216	443483	428175	339222	319597	71.15
P5R3	341921	338151	324891	263982	250637	73.30
P5R4	422453	417356	406965	339664	320241	75.81
P5R5	197597	195591	187040	152090	144756	73.26

* P1R5 and P5R1 were excluded prior to DNA extraction due to sample quality issues after transport.

Supplementary Table S4. Historical aridity descriptors (2000–2016) and field soil moisture at the five sampling sites.

Sampling Site	Mean annual AI	Pearson's *r*	Trend slope (AI units year^{-1})	*p*-value	95% CI of slope	Volumetric soil water content (%)
P1	0.220	0.488	0.0257	0.047	0.0004 to 0.0510	8.9–20.9
P2	0.406	0.444	0.0185	0.074	-0.0020 to 0.0390	5.0–11.7
P3	0.775	0.171	0.0039	0.511	-0.0085 to 0.0163	0.2–1.9; one replicate below the detection range
P4	0.843	0.075	0.0015	0.776	-0.0093 to 0.0122	10.2–25.3
P5	0.843	0.075	0.0015	0.776	-0.0093 to 0.0122	7.4–35.2

Note: AI, annual aridity index. Mean annual AI, Pearson's correlation coefficient (r), trend slope, p-value, and 95% confidence interval were calculated from annual AI values for the 2000–2016 period. Pearson's r describes the direction and strength of the temporal association between annual AI and time, whereas the trend slope represents the estimated annual rate of change in AI. Field soil moisture was measured independently during field sampling in 2025 and is reported as the observed range among replicates at each site.

Supplementary Table S5. Bacterial genera detected only in the pooled samples of each phytophysiognomic group.

Herbaceous–shrubland environment	Succulent shrubland environment	Agricultural environment
Acidithiobacillus	Fodinicola	Actibacterium
Actinobacillus	Microcoleus_SAG_1449-1a	Actinopolymorpha
Actinomyces	Planosporangium	Actinotalea
Actinospica	Scytonema_UTEX_2349	Acuticoccus
Aeromonas	Thermoleophilum	Adhaeribacter
Agathobacter	Thermosporothrix	Adlercreutzia
Alicyclobacillus	Wilmottia_Ant-Ph58	Agromyces
Alistipes		Albibacter
Alkanibacter		Algoriphagus
Amantichitinum		Aliterella
Aneurinibacillus		Alkaliphilus
Anoxybacillus		Alkanindiges
Bifidobacterium		Allocatelliglobosispora
Bilophila		Alloiococcus
Brachybacterium		Amaricoccus
Brevibacillus		Aminobacter
Cavicella		Anaerocolumna
Chamaesiphon_PCC-743		Anaerosporobacter
Coprococcus		Anaerovorax
Crenobacter		Aquabacterium
Dysgonomonas		Arboricoccus
Effusibacillus		Archangium
Endobacter		Arcticibacter
Enhydrobacter		Aureimonas
Estrella		Azohydromonas
Finegoldia		Azospirillum
Frankia		Azotobacter
Fusicatenibacter		Azovibrio
Gemella		Barnesiella
Geothrix		Belliella
Heliimonas		Blastopirellula
Intestinimonas		Brevundimonas
Kineococcus		Brochothrix
Lawsonella		Bythopirellula
Marinobacter		Caedibacter
Methanosaeta		Carnobacterium
Methylocapsa		Catellatospora
Methylorosula		Catenuloplanes
Methyloversatilis		Cellulosilyticum
Micropepsis		Cellulosimicrobium
Nevskia		Cereibacter

Occallatibacter
Oscillibacter
Oxalophagus
Parabacteroides
Parvibaculum
Pasteuria
Pedosphaera
Porphyromonas
Providencia
Psychroflexus
Rickettsiella
Risungbinella
Robiginitalea
Rudaea
Ruminiclostridium
Ruminococcus
Rummeliibacillus
Sediminibacterium
Sporomusa
Subdoligranulum
Terriglobus
Thiobacillus
Tyzzerella
Variibacter
Vicingus
Winogradskyella

Cesiribacter
Chiayiivirga
Chloronema
Chryseolinea
Chthonobacter
Chungangia
Citricoccus
Cnuella
Confluentibacter
Constrictibacter
Crocinitomix
Cryptosporangium
Curtobacterium
Dechloromonas
Desertibacter
Desertifilum_KR212-2
Desulfitobacterium
Dialister
Dietzia
Dinghuibacter
Domibacillus
Emticicia
Enterococcus
Euzebya
Exiguobacterium
Faecalibacterium
Falsirhodobacter
Ferrovibrio
Fictibacillus
Fluviicola
Fonticella
Frondihabitans
Fulvivirga
Geminicoccus
Georgenia
Glutamicibacter
Gottschalkia
Haemophilus
Hahella
Hamadaea
Ilumatobacter
Isosphaera
Jeotgalicoccus
Jiangella
Kallotenue
Kitasatospora

Kocuria
Kouleothrix
Laceyella
Larkinella
Leptolyngbya_EcFYyyy-
Leptospirillum
Leptothrix
Limibaculum
Limnothrix
Litorilinea
Longimicrobium
Luteimonas
Lutispora
Magnetospirillum
Malikia
Marvinbryantia
Methanosphaera
Microcoleus_PCC-7113
Monoglobus
Mumia
Myxococcus
Nannocystis
Neisseria
Niabella
Nibribacter
Nitratireductor
Nitrosomonas
Nodosilinea_PCC-714
Ochrobactrum
Olivibacter
Olsenella
Ornithinibacillus
Ornithinimicrobium
Oscillatoria_PCC-634
Oscillochloris
Paeniglutamicibacter
Paenisporosarcina
Pajaroellobacter
Paludisphaera
Panacagrimonas
Pantoea
Paracoccus
Parapedobacter
Parasegetibacter
Paucimonas
Paucisalibacillus

Pelagibius
Permianibacter
Phaeodactylibacter
Pilimelia
Piscinibacter
Planctomicrobium

Planktothrix_NIVA-CYA_15
Planococcus
Planomicrobium
Plasticicumulans
Pleurocapsa_PCC-7319
Polymorphobacter
Pontibacter
Promicromonospora
Prosthecomicrobium
Pseudactinotalea
Pseudofulvimonas
Pseudohongiella
Pseudorhizobium
Pseudosphingobacterium
Pseudoxanthobacter
Pusillimonas
Quinella
Rathayibacter
Rhabdobacter
Rhizorhapis
Rhodocytophaga
Rhodopseudomonas
Rhodovarius
Romboutsia
Roseimaritima
Roseobacter_clade_CHAB-I-5_lineage
Rubellimicrobium
Rubritepida
Rubrivirga
Rufibacter
Saccharothrix
Salinimicrobium
Salinispora
Sandaracinobacter
Sandarakinorhabdus
Sedimentibacter
Sediminicola
Shimazuella

Siccirubricoccus
Smaragdicoccus
Solitalea
Sphaerobacter
Sphingoaurantiacus
Sphingopyxis
Sphingorhabdus
Sporacetigenium
Stella
Stenotrophobacter
Syntrophobacter
Tagaea
Tahibacter
Taibaiella
Taonella
Terribacillus
Thermincola
Thermoflavimicrobium
Thermomonas
Tissierella
Tolypothrix
Trichococcus
Trichocoleus_SAG_26.92
Truepera
Tuwongella
Umezawaea
Urania-1B-19_marine_sediment_group
Verticiella
Vitellibacter
Vulgatibacter
Waddlia
Williamsia
Woeseia
Xanthobacter
Xanthomonas

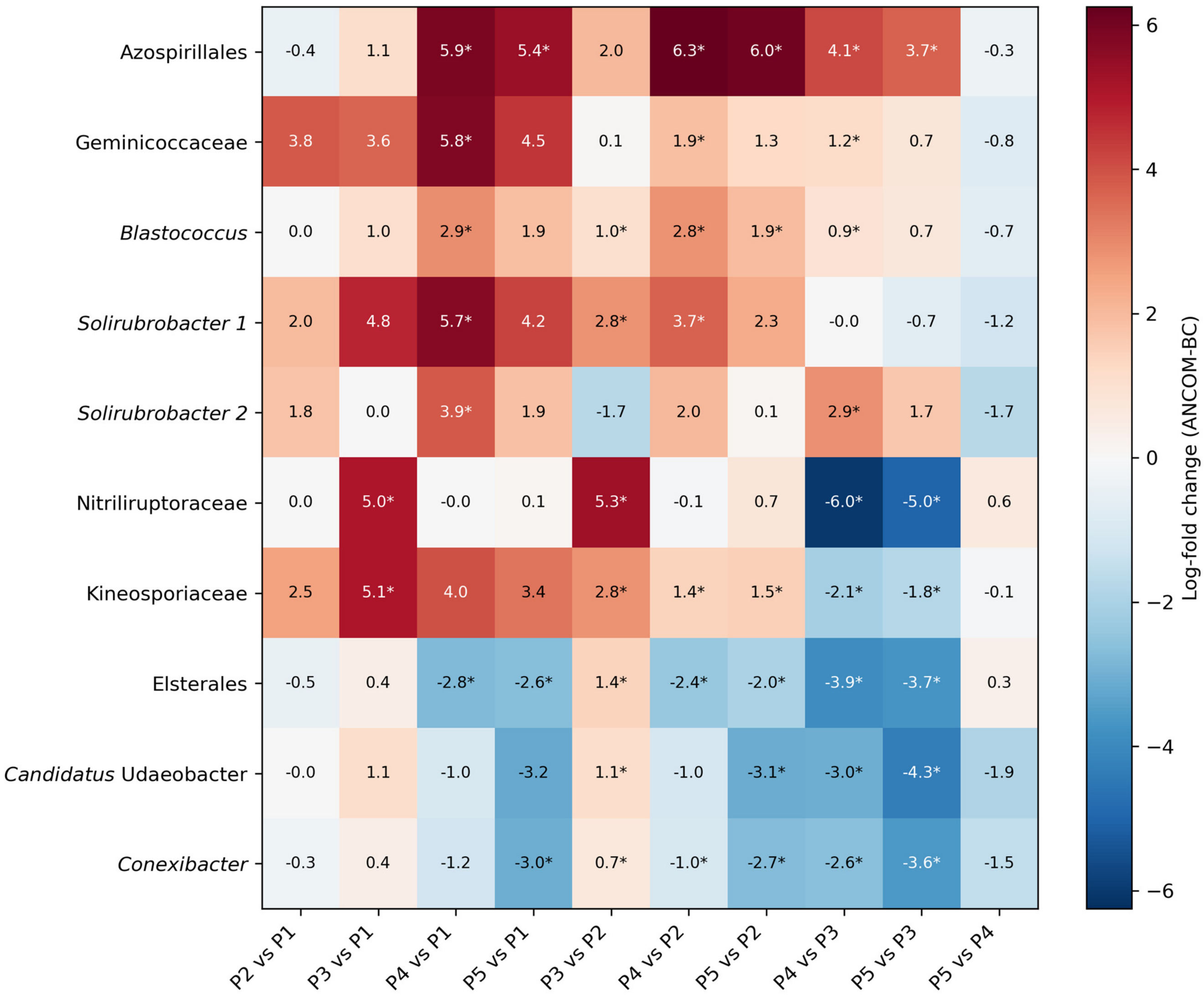

Supplementary Figure S1. Differential abundance of bacterial taxa between pairs of sampling sites, estimated with ANCOM-BC at the order, family, and genus levels. The taxa shown are those identified in the exploratory STAMP screening. Solirubrobacter 1 and 2 correspond to two lineages assigned to this genus in SILVA 138. Values represent log-fold changes for the first site relative to the second, and positive values (red) indicate higher abundance at the first site. Asterisks indicate significant differences (Holm-adjusted $q < 0.05$). P1 and P2, herbaceous–shrubland; P3, succulent shrubland; P4 and P5, agricultural.

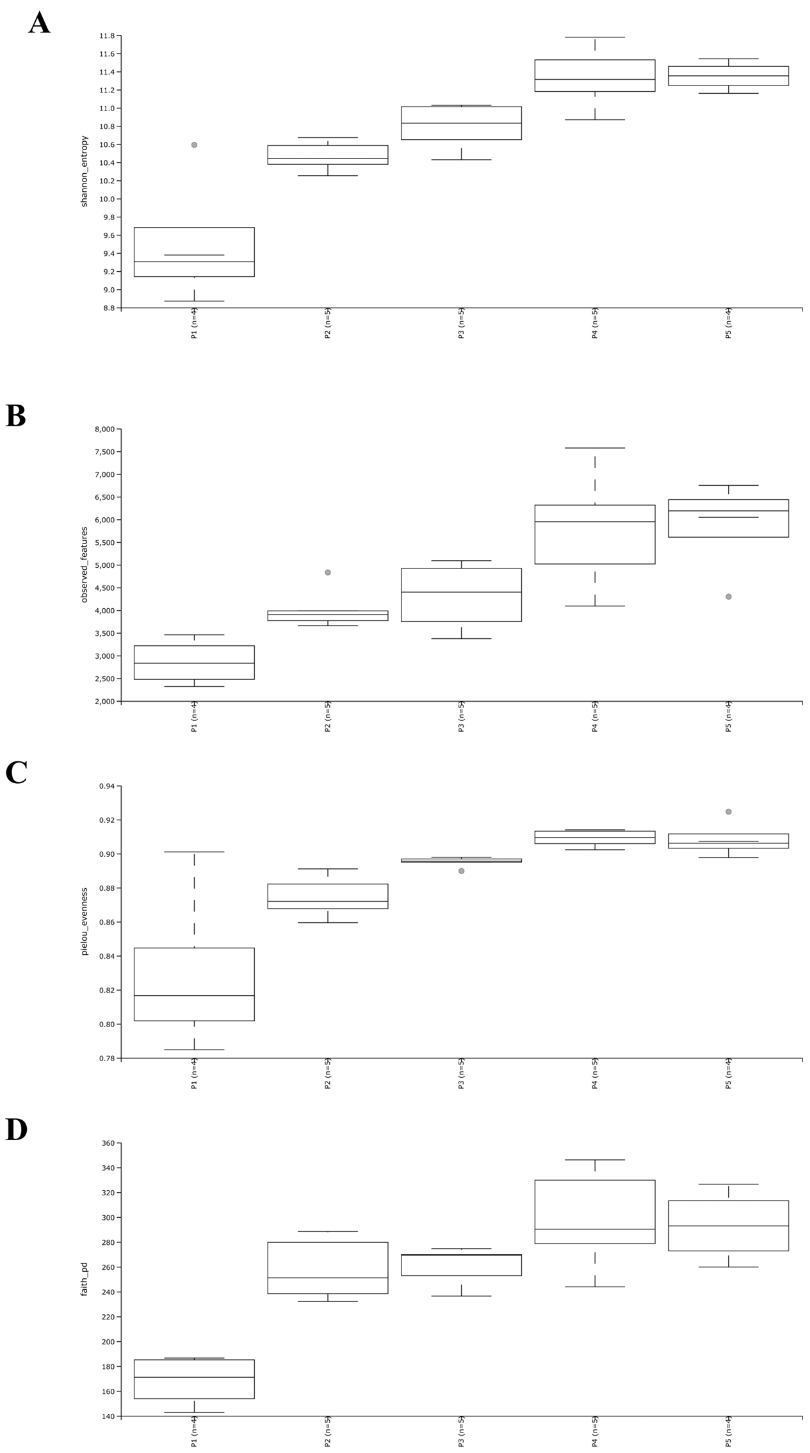

Supplementary Figure S2. Alpha diversity of soil bacterial communities at each sampling site. (A) Shannon diversity, (B) observed features, (C) Pielou's evenness, and (D) Faith's phylogenetic diversity. P1 and P2, herbaceous–shrubland; P3, succulent shrubland; P4 and P5, agricultural. Statistical comparisons among sites are presented in Table 3.

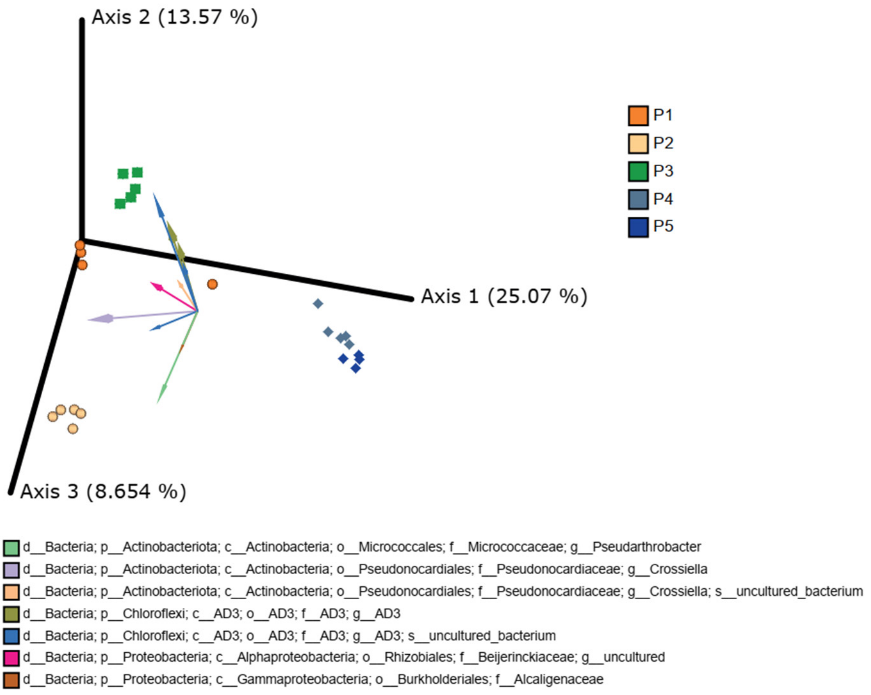

Supplementary Figure S3. Principal Coordinates Analysis (PCoA) based on unweighted UniFrac distances showing variation in soil bacterial community composition across the five sampling sites. Arrows represent the 10 bacterial taxa with the largest vector magnitudes in the ordination space. Arrow direction indicates the ordination gradient along which the relative sequence proportion of each taxon increases, whereas arrow length reflects the vector magnitude.

Supplementary Table S6. Global and pairwise PERMANOVA results based on unweighted UniFrac distances (999 permutations).

Global PERMANOVA

Grouping variable	Groups	Sample size	df	pseudo-F	p-value
Sampling site	5	23	4, 18	4.67	0.001

Pairwise PERMANOVA

Group 1	Group 2	Sample size	Permutations	pseudo-F	p-value	q-value
P1	P2	9	999	3.248357	0.004	0.01
	P3	9	999	4.302679	0.004	0.01
	P4	9	999	5.276034	0.009	0.015
	P5	8	999	5.640656	0.027	0.027
P2	P3	10	999	4.220599	0.004	0.01
	P4	10	999	5.549677	0.013	0.015556
	P5	9	999	5.976119	0.014	0.015556
P3	P4	10	999	4.87903	0.004	0.01
	P5	9	999	5.763992	0.013	0.015556
P4	P5	9	999	1.978505	0.006	0.012

p-values represent the raw pairwise test results, whereas q-values correspond to Benjamini–Hochberg-adjusted p-values for multiple comparisons.

Supplementary Table S7. Global and pairwise PERMDISP results based on unweighted UniFrac distances (999 permutations).

Global PERMDISP

Grouping variable	Groups	Sample size	df	F-value	p-value
Sampling site	5	23	4, 18	0.686	0.153

Pairwise PERMDISP

Group 1	Group 2	Sample size	Permutations	F-value	p-value	q-value
	P3	9	999	0.461217	0.152	0.291667
	P4	9	999	0.285856	0.275	0.392857
	P5	8	999	0.130111	0.413	0.51625
P2	P3	10	999	2.16543	0.027	0.0725
	P4	10	999	0.610032	0.029	0.0725
	P5	9	999	1.790613	0.007	0.07
P3	P4	10	999	0.017832	0.658	0.731111
	P5	9	999	8.182649	0.014	0.07
P4	P5	9	999	2.460269	0.175	0.291667

p-values represent the raw pairwise test results, whereas q-values correspond to Benjamini–Hochberg-adjusted p-values for multiple comparisons.
